# A new perspective on the functioning of the brain and the mechanisms behind conscious processes

**DOI:** 10.3389/fpsyg.2013.00242

**Published:** 2013-04-30

**Authors:** Joachim Keppler

**Affiliations:** DIWISSRoth, Germany

**Keywords:** consciousness, quantum physics, stochastic electrodynamics, zero-point field, attractors

## Abstract

An essential prerequisite for the development of a theory of consciousness is the clarification of the fundamental mechanisms underlying conscious processes. In this article I present an approach that sheds new light on these mechanisms. This approach builds on stochastic electrodynamics (SED), a promising theoretical framework that provides a deeper understanding of quantum systems and reveals the origin of quantum phenomena. I outline the most important concepts and findings of SED and interpret the neurophysiological body of evidence in the context of these findings, indicating that the functioning of the brain rests upon exactly the same principles that are characteristic for quantum systems. On this basis, I construct a new hypothesis on the mechanisms behind conscious processes and discuss the new perspectives this hypothesis opens up for consciousness research. In particular, it offers the possibility of elucidating the relationship between brain and consciousness, of specifying the connection between consciousness and information, and of answering the question of what distinguishes conscious processes from unconscious processes.

## Introduction

Although the neurosciences have made considerable advances in the last two decades, the core problem of how brain processes cause consciousness is still outstanding. Since the fundamental mechanisms of the physical world are described on the basis of quantum field theories, the question arises whether quantum physics is also relevant for the understanding of consciousness. As far as this question is concerned, the scientific community is divided by a gulf separating those who regard the properties of quantum systems as essential ingredients of a theory of consciousness (Beck and Eccles, 1992; Stapp, 1993; Hameroff and Penrose, 1996) from those who cast doubt on a deeper connection between quantum physics and consciousness and, hence, contest the necessity of quantum approaches to consciousness (Tegmark, 2000; Koch and Hepp, 2006; Baars and Edelman, 2012; Seth, 2012).

Where does the disparity of opinions come from? On closer inspection, it becomes obvious that the discrepancy originates mainly from the unsatisfactory overall situation of quantum theory. Although the formalism of quantum physics is highly developed and all the predictions are perfectly confirmed, the explanatory approaches to quantum phenomena leave many questions unresolved. This is the cause for a whole lot of misinterpretations and misunderstandings regarding the nature of quantum systems and the conditions under which they can develop.

In order to bridge the divide and appreciate the real value of quantum physics for consciousness research, we need more transparency about the processes that take place in the quantum world. A small circle of physicists accepted this challenge and strived after concepts that eliminate the explanatory gaps of quantum theory. The most developed approach is stochastic electrodynamics (SED), which has achieved significant progress over the years. Instead of purely describing quantum phenomena, as the established formalism of quantum physics does, SED provides a look behind the scenes of quantum systems, thereby disclosing the fundamental principles underlying matter, explaining the origin of quantum phenomena and unraveling the mysteries of quantum physics.

This article is grounded on the assumption that SED is the proper foundation for the understanding of quantum systems. I outline the central insights of SED and interpret the neurophysiological body of evidence in the context of these insights. On this basis, I construct a new hypothesis on the functioning of the brain and the mechanisms behind conscious processes. Finally, I discuss the new perspectives this hypothesis opens up for consciousness research.

## Fundamental mechanisms underlying quantum systems

The foundations of SED were established in the 1960's and 1970's (Marshall, 1963, 1965; Boyer, 1969, 1975) with the goal to derive the laws of quantum physics from first principles. Since then, the framework of SED has been continuously advanced, most notably by important theoretical works (De la Peña and Cetto, 1994, 1995, 1996, 2001, 2006) and insightful numerical simulations (Cole and Zou, 2003, 2004a,b). Through the recent developments many of the initial problems could be resolved, now enabling the derivation of the full formalism of quantum mechanics and quantum electrodynamics from SED (De la Peña and Cetto, 2001).

SED is based on the conception that the vacuum is imbued with a real, all-pervasive stochastic radiation field, called zero-point field (ZPF), which may be viewed as an ocean of energy that permeates the whole universe, making the vacuum in reality a plenum. The undisturbed ZPF exhibits several symmetries, namely homogeneity, isotropy, Lorentz invariance, and scale invariance, and can be described as a sum of plane electromagnetic waves with random phase and a characteristic power spectrum. In this form, the ZPF constitutes a background of permanent activity that is present even at absolute zero.

According to SED, the components of every physical system interact permanently and unavoidably with the ZPF, thus acquiring a stochastic motion and behaving as stochastic oscillators. As long as a system is sufficiently shielded against thermal noise and the ZPF is the dominating driving force, the energy exchange between the system components and the ZPF can reach equilibrium states where the average power absorbed by the system compensates exactly the average radiated power. These balance situations are identical with the stationary states predicted by quantum theory (De la Peña and Cetto, 2001, 2006). Hence, any dynamical system in balance with the ZPF displays quantum behavior. Upon reaching equilibrium, such a system falls into a stable attractor (De la Peña and Cetto, 1995).

Since matter and ZPF exert a mutual influence, the presence of matter also affects the dynamics of the ZPF. In the case of a nonlinear system in equilibrium, the interaction between the system components and the ZPF leads to a modification of the ZPF. As soon as a stable attractor is reached, the frequency components involved in the maintenance of the equilibrium can become highly correlated (De la Peña and Cetto, 2001), resulting in a de-randomization and partial organization of the local field (De la Peña et al., 2009). This means that the formation of an attractor imprints a system-specific information state on the ZPF. In this way, all the components of the system are effectively coupled through the ZPF, giving rise to collective cooperation and long-range coherence (De la Peña and Cetto, 2001). Hence, the key insight from SED is that the properties of quantum systems are emergent phenomena that can be traced back to the resonant interaction between the system components and the ZPF. So, let us register: *A quantum system functions as a resonant stochastic oscillator that selectively filters its specific resonance frequencies out of the ZPF spectrum. As soon as a quantum system reaches a stable attractor, the system components display long-range coherence, which is mediated by the information-bearing, partially organized ZPF*.

Given these universal mechanisms, it is quite obvious that there is no clear separation between the microcosm and the macrocosm. Independent of the system size, it is always the resonant coupling of the system components to specific frequencies of the ZPF spectrum that enables pattern formation and leads to quantum behavior. On small length scales pattern formation is facilitated by the higher frequencies of the ZPF spectrum, for larger and larger systems the lower frequencies get more and more important. Thus, it is to be expected that under appropriate conditions, primarily neutralization of disruptive thermal effects, quantum phenomena can arise in many macroscopic systems, particularly in biological systems (Lloyd, 2011).

## Fundamental mechanisms in the brain

In order to judge whether or not the brain is a quantum system, we have a look at the body of evidence resulting from the analysis of neural activity patterns. A common strategy behind these research activities, which have been continuously improved and methodologically refined over the years (Aru et al., 2012), consists in distilling the neural correlates of consciousness (NCC).

To begin with, it is widely accepted that consciousness is associated with long-range coherence in the brain, particularly with synchronized activity in the gamma frequency band (Crick and Koch, 1990; Desmedt and Tomberg, 1994; Engel and Singer, 2001; Melloni et al., 2007). In more detail, new results suggest that “discrete moments of perceptual experience are implemented by transient gamma-band synchronization of relevant cortical regions, and that disintegration and reintegration of these assemblies is time-locked to ongoing theta oscillations” (Doesburg et al., 2009). Moreover, it was found that gamma synchrony shows up not only during attention to an external stimulus, but also in altered states of consciousness, such as meditation (Lutz et al., 2004) and REM sleep (Llinás and Ribary, 1993; Montgomery et al., 2008).

As for the characteristics of the gamma oscillations, a time-frequency analysis of the local field potentials (LFP) revealed that “the source of gamma-band peaks is of stochastic nature” (Burns et al., 2010) and that “gamma activity is indistinguishable from filtered noise” (Burns et al., 2011). Hence, gamma activity cannot be understood on the basis of deterministic network models. Rather, noise seems to play an essential role in the generation of gamma synchrony, so that in a realistic model the brain should be “viewed as a resonant stochastic oscillator” (Burns et al., 2010). Furthermore, also experiments investigating stochastic resonance (SR) within and between brain areas imply that “SR-mediated neural synchronization is a general mechanism of brain functioning” (Ward et al., 2006) and that “noise could play a fundamental role in biological information processing” (Kitajo et al., 2007).

The analysis of EEG and MEG background activity showed that spontaneous oscillations in the brain exhibit 1/f power-law scaling behavior, indicating that the brain operates in a scale-free state of self-organized criticality (Linkenkaer-Hansen et al., 2001; Freeman et al., 2003). Such scaling behavior was also verified for the LFP in humans (Milstein et al., 2009). It is quite revealing that the origin of this behavior can be explained as a quantum phenomenon involving the ZPF (Cavalleri and Bosi, 2007).

I would like to complete the compilation of evidence with the findings of Walter Freeman. His studies in animals showed that conditioned stimuli are associated with macroscopic patterns of amplitude modulation of a carrier wave in the gamma and beta frequency range, which represent attractors in an attractor landscape (Freeman, 1991, 2005). The results further suggest that these attractors are the NCC, since the corresponding activity patterns are “not fixed representations of the stimuli,” but rather are “correlated with the actions and inferred perceptions of the animals” (Freeman, 2007). It was discovered that “vast collections of neurons shift abruptly and simultaneously” between different attractors (Freeman, 1991), resembling “cinematographic frames” whose “rapid and efficient formation and dissolution” can only be understood on the basis of many-body quantum theory (Freeman and Vitiello, 2006). From this perspective, the brain can be regarded as a complex system that operates near a critical point of a phase transition. While displaying spontaneous activity and irregular dynamics in the disordered phase, an appropriate stimulus can transfer the brain to the ordered phase that exhibits long-range correlations and stable attractors. These features cannot be explained without recourse to quantum dynamics (Freeman and Vitiello, 2006).

Taken as a whole, the currently available body of evidence and the entirety of observations suggest that the brain has all the characteristics of a macroscopic quantum system. This becomes particularly obvious when we interpret the neurophysiological findings in the context of SED and the consequent organizing principles summarized in the previous section. Accordingly, the brain can be viewed as a resonant stochastic oscillator driven by the ZPF, which acts as a ubiquitous noise source causing the spontaneous background activity in the disordered phase of the brain. A suitable sensory input induces a transition to the ordered phase and prompts a cell assembly of the brain to fall into an attractor. As soon as a stationary state is reached, the assembly enters the quantum regime, displays long-range coherence and imprints a specific information state on the local ZPF. In the regular perceptual process such phase transitions are repeated with frequencies in the theta range. A better understanding of this process, which includes all the levels of microscopic and macroscopic organization, is still required, with special attention to the conditions under which long-range coherence is possible. The latest studies point to the importance of interfacial water that effectively neutralizes thermal disturbing effects and facilitates coherence (Del Giudice et al., 2005, 2010).

It can be concluded that the brain evidently provides an environment that is sufficiently shielded against thermal noise, making the ZPF the dominating agent and communication medium that orchestrates the brain activity and enables the emergence of quantum phenomena. This indicates that *the recurrent formation and dissolution of quantum states constitutes a fundamental mechanism in the brain*. Whenever the activity of the brain reaches a stable attractor, a ZPF information state is generated and a conscious experience arises.

## Brain and consciousness

In order to define the relationship between brain and consciousness still more precisely, additional input is required. In my approach, I take this input from Eastern philosophy, a discipline that has a long tradition in studying consciousness very systematically. A detailed comparison between the findings of SED and the insights of Eastern philosophy reveals not only a striking congruence as far as the basic principles behind matter are concerned. It also gives us the important hint that the ZPF is a promising candidate for the carrier of consciousness, suggesting that *consciousness is a fundamental property of the universe*, that *the ZPF is the substrate of consciousness* and that *our individual consciousness is the result of a dynamic interaction process that causes the realization of ZPF information states* (Keppler, 2012).

**Figure 1:**
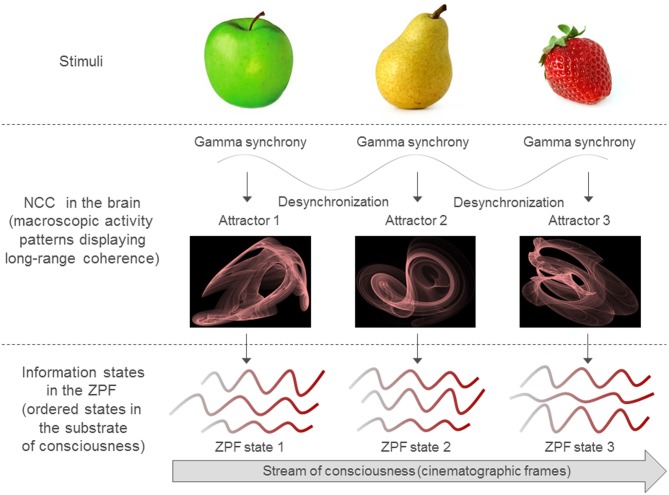
**The brain produces a stream of consciousness by periodically modifying the ZPF and generating ZPF information states.** This process is triggered by appropriate stimuli that induce macroscopic patterns of synchronized activity in the gamma frequency band. These long-range activity patterns, which represent attractors in an attractor landscape, are the NCC. In the regular perceptual process the alternation of synchronization and desynchronization is linked to theta oscillations. Whenever the brain activity falls into a stable attractor, there is a corresponding ZPF information state, which carries the integrated information of the attractor and is characterized by specific correlations between frequency components of the ZPF spectrum. Every ZPF information state is associated with a conscious state, i.e., every ordered pattern in ZPF information space corresponds to a phenomenal state in qualia space.

These hypotheses express that consciousness as such is not produced by matter. Rather, they imply that matter and consciousness have a common basis in the ZPF, which not only orchestrates matter and gives birth to the enormous variety of phenomena in our physical world, but also forms the foundation of our conscious experiences. In that it is ubiquitous and equipped with unique properties, the ZPF has the potential to define a universally standardized substratum for our conscious minds, giving rise to the conjecture that *the brain is a complex instrument that filters the varied shades of sensations and emotions selectively out of the all-pervasive field of consciousness, the ZPF*. This is achieved by the fundamental mechanism described above, which leads to sequences of attractors. Every attractor acts as a frequency filter on the ZPF and generates a characteristic frequency pattern, thus specifying a ZPF information state that is associated with a conscious state. In this way *the brain produces an individual stream of consciousness by periodically modifying the ZPF and generating ZPF information states*. This process is illustrated in Figure 1.

## Discussion and concluding remarks

The SED-based approach addresses a core problem of current neuroscience and casts new light on the mechanisms underlying conscious experience. In this way, it opens a door to the transition from correlation to explanation, which “requires an understanding of *why* particular NCCs have a privileged relationship with consciousness” (Seth, 2009). The crucial point is that only attractors, which are synchronized activity patterns orchestrated by the ZPF, provide access to the substrate of consciousness to such an extent that they leave fingerprints in this substrate. Hence, conscious processes can be distinguished from unconscious processes in that only the former processes bring about activity patterns that reach equilibrium with the ZPF and are accompanied by ZPF information states. Depending on the dynamic situation, the same assemblies of a network can be involved in conscious and unconscious processes.

In the wider context of the cognitive cycle every conscious process is preceded by a set of preconscious processes that are potentially capable of entering consciousness (Dehaene et al., 2006; Franklin and Baars, 2010). According to the ideas brought forth in this article, none of the competing activity patterns representing the preconscious processes is a fully developed attractor. Rather, these activity patterns are attractors in early stages of development, which display some of the dynamical characteristics of fully developed attractors but have not exceeded the required equilibration time and reached equilibrium with the ZPF. Therefore, in the preconscious phase no ZPF information state is generated and no conscious experience arises. Only one selected and attentionally amplified activity pattern gets the chance to fully unfold and develop to a full-blown attractor that reaches equilibrium with the ZPF, generates a ZPF information state and results in a conscious moment.

The concept of ZPF information states is totally in line with the double-aspect principle of information, which is a “candidate for a basic principle that might form the cornerstone of a fundamental theory of consciousness” (Chalmers, 1995). Moreover, the SED-based approach specifies this principle in that it imposes a constraint on the sort of information that is associated with consciousness. Based on the hypothesis that the ZPF is the carrier of consciousness, only nonlinear systems that interact dynamically with the ZPF and generate ZPF information states can produce conscious states. The internal aspects of such information states are phenomenal, i.e., a conscious moment is a ZPF information state experienced from inside. The external aspects of such information states are physical and show up as NCC (Keppler, 2012).

Taken to its logical end, the SED-based approach indicates that all microscopic and macroscopic quantum systems may be conscious, with the quantity and quality of consciousness being associated with the complexity of the system. While simple systems, which are characterized by relatively sparse attractor landscapes and relatively simple attractors, are endowed with a rudimentary form of consciousness, complex systems have very rich and highly adaptive attractor landscapes with complex attractors, giving rise to a broad spectrum of conscious experiences. These concepts are related to the integrated information theory (Tononi, 2004, 2008) and similar approaches that measure consciousness by means of dynamical complexity (Seth, 2009). The strong point of the SED framework is that it is able to underlay these approaches with a universal dynamical mechanism of information integration and, hence, specifies how and where the integration takes place. The key finding is that the integration cannot be accomplished by the network architecture alone. Rather, it requires the ZPF as an integrating agent and it is the ordered ZPF configuration behind an attractor that carries the integrated information.

In summary, the presented approach is fully consistent with the findings of physics, neuroscience, and Eastern philosophy. Instead of tying consciousness completely and utterly to the material structure of the brain, the approach suggests that the universe is imbued with an all-pervasive substrate of consciousness and explains how the brain shapes this substrate in a causally closed functional chain, thus opening up entirely new perspectives for consciousness research. The next step consists in making testable predictions. This will involve a high degree of physics and means that we have to build SED-based oscillator models of the brain that are sufficiently realistic in order to reproduce the observed attractor dynamics. When such models are in place, we can pass through a number of conscious states, identify the attractors, determine the corresponding ZPF information states and systematically classify ZPF information space on the basis of the first-person accounts (Keppler, 2012). This will bring us closer to the goal of understanding how exactly the human brain produces a stream of phenomenal consciousness.

### Conflict of interest statement

The author declares that the research was conducted in the absence of any commercial or financial relationships that could be construed as a potential conflict of interest.

## References

[B1] Aru J. Bachmann T. Singer W. Melloni L. (2012). Distilling the neural correlates of consciousness. Neurosci. Biobehav. Rev. 36, 737–746 10.1016/j.neubiorev.2011.12.003 22192881

[B2] Baars B. J. Edelman D. B. (2012). Consciousness, biology and quantum hypotheses. Phys. Life Rev. 9, 285–294 10.1016/j.plrev.2012.07.001 22925839

[B3] Beck F. Eccles J. C. (1992). Quantum aspects of brain activity and the role of consciousness. Proc. Natl. Acad. Sci. U.S.A. 89, 11357–11361 1333607 10.1073/pnas.89.23.11357 PMC50549

[B4] Boyer T. H. (1969). Derivation of the blackbody radiation spectrum without quantum assumptions. Phys. Rev. 182, 1374–1383

[B5] Boyer T. H. (1975). Random electrodynamics: the theory of classical electrodynamics with classical electromagnetic zero-point radiation. Phys. Rev. D 11, 790–808

[B6] Burns S. P. Xing D. Shapley R. M. (2011). Is gamma-band activity in the local field potential of V1 cortex a “clock” or filtered noise? J. Neurosci. 31, 9658–9664 10.1523/JNEUROSCI.0660-11.2011 21715631 PMC3518456

[B7] Burns S. P. Xing D. Shelley M. J. Shapley R. M. (2010). Searching for autocoherence in the cortical network with a time-frequency analysis of the local field potential. J. Neurosci. 30, 4033–4047 10.1523/JNEUROSCI.5319-09.2010 20237274 PMC2897248

[B8] Cavalleri G. Bosi L. (2007). Origin of 1/f noise as a runaway phenomenon due to the zero-point field (ZPF) of quantum electrodynamics (QED). Phys. Stat. Sol. C 4, 1230–1233

[B9] Chalmers D. J. (1995). Facing up to the problem of consciousness. J. Conscious. Stud. 2, 200–219 2816553

[B10] Cole D. C. Zou Y. (2003). Quantum mechanical ground state of hydrogen obtained from classical electrodynamics. Phys. Lett. A 317, 14–20

[B11] Cole D. C. Zou Y. (2004a). Simulation study of aspects of the classical hydrogen atom interacting with electromagnetic radiation: circular orbits. J. Sci. Comput. 20, 43–68 14995730

[B12] Cole D. C. Zou Y. (2004b). Simulation study of aspects of the classical hydrogen atom interacting with electromagnetic radiation: elliptical orbits. J. Sci. Comput. 20, 379–404 14995730

[B13] Crick F. Koch C. (1990). Towards a neurobiological theory of consciousness. Sem. Neurosci. 2, 263–275

[B14] Dehaene S. Changeux J.-P. Naccache L. Sackur J. Sergent C. (2006). Conscious, preconscious, and subliminal processing: a testable taxonomy. Trends Cogn. Sci. 10, 204–211 10.1016/j.tics.2006.03.007 16603406

[B15] De la Peña L. Cetto A. M. (1994). Quantum phenomena and the zeropoint radiation field. Found. Phys. 24, 917–948 10.1021/nl051007n 16089492

[B16] De la Peña L. Cetto A. M. (1995). Quantum phenomena and the zeropoint radiation field II. Found. Phys. 25, 573–604

[B17] De la Peña L. Cetto A. M. (1996). The Quantum Dice: An Introduction to Stochastic Electrodynamics. Dordrecht: Kluwer Academic Publishers

[B18] De la Peña L. Cetto A. M. (2001). Quantum theory and linear stochastic electrodynamics. Found. Phys. 31, 1703–1731

[B19] De la Peña L. Cetto A. M. (2006). The foundations of linear stochastic electrodynamics. Found. Phys. 36, 350–368

[B20] De la Peña L. Valdés-Hernández A. Cetto A. M. (2009). Quantum mechanics as an emergent property of ergodic systems embedded in the zero-point radiation field. Found. Phys. 39, 1240–1272

[B21] Del Giudice E. De Ninno A. Fleischmann M. Mengoli G. Milani M. Talpo G. (2005). Coherent quantum electrodynamics in living matter. Electromagn. Biol. Med. 24, 199–210

[B22] Del Giudice E. Spinetti P. R. Tedeschi A. (2010). Water dynamics at the root of metamorphosis in living organisms. Water 2, 566–586

[B23] Desmedt J. E. Tomberg C. (1994). Transient phase-locking of 40 Hz electrical oscillations in prefrontal parietal cortex reflects the process of conscious somatic perception. Neurosci. Lett. 168, 126–129 8028764 10.1016/0304-3940(94)90432-4

[B24] Doesburg S. M. Green J. J. McDonald J. J. Ward L. M. (2009). Rhythms of consciousness: binocular rivalry reveals large-scale oscillatory network dynamics mediating visual perception. PLoS ONE 4:e6142 10.1371/journal.pone.0006142 19582165 PMC2702101

[B25] Engel A. K. Singer W. (2001). Temporal binding and the neural correlates of sensory awareness. Trends Cogn. Sci. 5, 16–25 10.1016/S1364-6613(00)01568-0 11164732

[B26] Franklin S. Baars B. (2010). Two varieties of unconscious processes, in New Horizons in the Neuroscience of Consciousness, eds Perry E. Collerton D. Ashton H. LeBeau F. (Amsterdam: John Benjamin), 91–102

[B27] Freeman W. J. (1991). The physiology of perception. Sci. Am. 264, 78–85 2000483 10.1038/scientificamerican0291-78

[B28] Freeman W. J. (2005). Origin, structure, and role of background EEG activity. Part 3. Neural frame classification. Clin. Neurophysiol. 116, 1118–1129 10.1016/j.clinph.2004.12.023 15826853

[B29] Freeman W. J. (2007). Indirect biological measures of consciousness from field studies of brains as dynamical systems. Neural Netw. 20, 1021–1031 10.1016/j.neunet.2007.09.004 17923391

[B30] Freeman W. J. Holmes M. D. Burke B. C. Vanhatalo S. (2003). Spatial spectra of scalp EEG and EMG from awake humans. Clin. Neurophysiol. 114, 1053–1068 10.1016/S1388-2457(03)00045-2 12804674

[B31] Freeman W. J. Vitiello G. (2006). Nonlinear brain dynamics as macroscopic manifestation of underlying many-body field dynamics. Phys. Life Rev. 3, 93–118

[B32] Hameroff S. R. Penrose R. (1996). Conscious events as orchestrated space-time selections. J. Conscious. Stud. 3, 36–53

[B33] Keppler J. (2012). A conceptual framework for consciousness based on a deep understanding of matter. Philos. Study 2, 689–703

[B34] Kitajo K. Doesburg S. M. Yamanaka K. Nozaki D. Ward L. M. Yamamoto Y. (2007). Noise-induced large-scale phase synchronization of human-brain activity associated with behavioural stochastic resonance. Europhys. Lett. 80:40009 10.1209/0295-5075/80/40009

[B35] Koch C. Hepp K. (2006). Quantum mechanics in the brain. Nature 440, 611–612 10.1038/440611a 16572152

[B36] Linkenkaer-Hansen K. Nikouline V. V. Palva J. M. Ilmoniemi R. J. (2001). Long-range temporal correlations and scaling behavior in human brain oscillations. J. Neurosci. 21, 1370–1377 11160408 10.1523/JNEUROSCI.21-04-01370.2001 PMC6762238

[B37] Llinás R. Ribary U. (1993). Coherent 40-Hz oscillation characterizes dream state in humans. Proc. Natl. Acad. Sci. U.S.A. 90, 2078–2081 10.1073/pnas.90.5.2078 8446632 PMC46024

[B38] Lloyd S. (2011). Quantum coherence in biological systems. J. Phys. Conf. Ser. 312:012037 10.1088/1742-6596/302/1/012037

[B39] Lutz A. Greischar L. L. Rawlings N. B. Ricard M. Davidson R. J. (2004). Long-term meditators self-induce high-amplitude gamma synchrony during mental practice. Proc. Natl. Acad. Sci. U.S.A. 101, 16369–16373 10.1073/pnas.0407401101 15534199 PMC526201

[B40] Marshall T. W. (1963). Random electrodynamics. Proc. R. Soc. Lond. A 276, 475–491

[B41] Marshall T. W. (1965). Statistical electrodynamics. Proc. Camb. Philos. Soc. 61, 537–546

[B42] Melloni L. Molina C. Pena M. Torres D. Singer W. Rodriguez E. (2007). Synchronization of neural activity across cortical areas correlates with conscious perception. J. Neurosci. 27, 2858–2865 10.1523/JNEUROSCI.4623-06.2007 17360907 PMC6672558

[B43] Milstein J. Mormann F. Fried I. Koch C. (2009). Neuronal shot noise and Brownian 1/f^2^ behavior in the local field potential. PLoS ONE 4:e4338 10.1371/journal.pone.0004338 19190760 PMC2629847

[B44] Montgomery S. M. Sirota A. Buzsáki G. (2008). Theta and gamma coordination of hippocampal networks during waking and REM sleep. J. Neurosci. 28, 6731–6741 10.1523/JNEUROSCI.1227-08.2008 18579747 PMC2596978

[B45] Seth A. K. (2009). Explanatory correlates of consciousness: theoretical and computational challenges. Cogn. Comput. 1, 50–63

[B46] Seth A. K. (2012). Putting Descartes before the horse: quantum theories of consciousness. Phys. Life Rev. 9, 297–298 10.1016/j.plrev.2012.07.005 22831961

[B47] Stapp H. P. (1993). A quantum theory of the mind-brain interface, in Mind, Matter, and Quantum Mechanics, ed Stapp H. P. (Berlin: Springer), 145–172

[B48] Tegmark M. (2000). The importance of quantum decoherence in brain processes. Phys. Rev. E 61, 4194–4206 10.1103/PhysRevE.61.4194 11088215

[B49] Tononi G. (2004). An information integration theory of consciousness. BMC Neurosci. 5:42 10.1186/1471-2202-5-42 15522121 PMC543470

[B50] Tononi G. (2008). Consciousness as integrated information: a provisional manifesto. Biol. Bull. 215, 216–242 19098144 10.2307/25470707

[B51] Ward L. M. MacLean S. E. Kirschner A. (2006). Stochastic resonance modulates neural synchronization within and between cortical sources. PLoS ONE 5:e14371 10.1371/journal.pone.0014371 21179552 PMC3002936

